# Asymmetrical Reinforcement and *Wolbachia* Infection in Drosophila

**DOI:** 10.1371/journal.pbio.0040325

**Published:** 2006-10-10

**Authors:** John Jaenike, Kelly A Dyer, Chad Cornish, Miranda S Minhas

**Affiliations:** 1 Department of Biology, University of Rochester, Rochester, New York, United States of America; 2 Institute of Evolutionary Biology, University of Edinburgh, Edinburgh, United Kingdom; Duke University, United States of America

## Abstract

Reinforcement refers to the evolution of increased mating discrimination against heterospecific individuals in zones of geographic overlap and can be considered a final stage in the speciation process. One the factors that may affect reinforcement is the degree to which hybrid matings result in the permanent loss of genes from a species' gene pool. Matings between females of Drosophila subquinaria and males of D. recens result in high levels of offspring mortality, due to interspecific cytoplasmic incompatibility caused by *Wolbachia* infection of D. recens. Such hybrid inviability is not manifested in matings between D. recens females and D. subquinaria males. Here we ask whether the asymmetrical hybrid inviability is associated with a corresponding asymmetry in the level of reinforcement. The geographic ranges of D. recens and D. subquinaria were found to overlap across a broad belt of boreal forest in central Canada. Females of *D. subquinaria* from the zone of sympatry exhibit much stronger levels of discrimination against males of *D. recens* than do females from allopatric populations. In contrast, such reproductive character displacement is not evident in *D. recens*, consistent with the expected effects of unidirectional cytoplasmic incompatibility. Furthermore, there is substantial behavioral isolation within D. subquinaria, because females from populations sympatric with D. recens discriminate against allopatric conspecific males, whereas females from populations allopatric with D. recens show no discrimination against any conspecific males. Patterns of general genetic differentiation among populations are not consistent with patterns of behavioral discrimination, which suggests that the behavioral isolation within D. subquinaria results from selection against mating with *Wolbachia*-infected D. recens. Interspecific cytoplasmic incompatibility may contribute not only to post-mating isolation, an effect already widely recognized, but also to reinforcement, particularly in the uninfected species. The resulting reproductive character displacement not only increases behavioral isolation from the *Wolbachia*-infected species, but may also lead to behavioral isolation between populations of the uninfected species. Given the widespread occurrence of *Wolbachia* among insects, it thus appears that there are multiple ways by which these endosymbionts may directly and indirectly contribute to reproductive isolation and speciation.

## Abstract 2

Interspecific cytoplasmic incompatibility caused by*Wolbachia* infection can contribute not only to post-mating isolation, but also to reinforcement, particularly in uninfected species, and therefore helps to promote reproductive isolation.

## Introduction

When incipient species come into secondary contact, natural selection against the production of unfit hybrids may favor the evolution of increased behavioral isolation between them, a process termed reinforcement [1,2]. Serious questions about the reality of reinforcement have been raised [3], but there is now good empirical evidence as well as a convincing theoretical foundation for reinforcement [4,5]. One possible outcome of the process of reinforcement is a pattern of reproductive character displacement, in which behavioral isolation is greater between individuals obtained from sympatric populations of two species than between individuals obtained from allopatric populations [3,6,7]. Reinforcement is typically modeled as acting symmetrically between species with partially overlapping ranges. However, the process may affect the two species differently if the range of one species is completely nested inside that of another [8], if levels of gene flow into the zone of sympatry differ between the species, or if there are differences in the fitness of reciprocal F1 hybrids.

One possible cause of unequal hybrid fitness is unidirectional cytoplasmic incompatibility (CI) induced by *Wolbachia* infection in one of the two species [9]. CI can occur in crosses between *Wolbachia*-infected males and uninfected females and results in high levels of embryonic mortality among the offspring. In the reciprocal cross between uninfected males and infected females, offspring survival is not affected. Thus, if one species in a zone of sympatry is infected with *Wolbachia* and the other is not, a large difference in the number of surviving offspring produced in reciprocal crosses between species may result.

Several aspects of *Wolbachia* biology suggest that *Wolbachia* could be a common cause of such asymmetry in insects. *Wolbachia* are widely distributed across insect species, where infection is most often manifested as CI [10,11]. Phylogenetic analyses indicate that infections are generally short-lived on an evolutionary time scale, so that sister insect taxa, including those that are only partially isolated, can differ in their infection status [12–14]. Although *Wolbachia* spread and are maintained as a result of the CI that they cause within species, the same phenomenon can be manifested in matings between species [13,15,16]. Consequently, there may be numerous pairs of partially isolated insect taxa between which there is asymmetrical interspecific CI.

Because CI leads to the death of offspring produced by matings between individuals that differ in infection status, there has been considerable interest in the role that *Wolbachia* may play in speciation [17,18]. Despite the appeal of this apparently simple isolating mechanism, there are substantial difficulties with speciation based solely on *Wolbachia*-induced CI [5,19]. From a theoretical perspective, CI causes unstable equilibrium in the prevalence of *Wolbachia* infection, rather than stable persistence of infected and uninfected populations [20,21]. Even bidirectional incompatibility entails an unstable equilibrium, with only one type expected at equilibrium [22]. Theoretical analyses also indicate that the spread of *Wolbachia* throughout a continuous population will occur much more rapidly than evolution of reproductive isolation [23]. Empirically, there is no evidence that *Wolbachia* contributes to hybrid sterility or to Haldane's rule, two of the most general features of animal speciation [5]. These findings indicate that *Wolbachia* cannot be the primary cause of many speciation events.

Nevertheless, *Wolbachia* may contribute, along with other mechanisms, to overall isolation between populations [13]. In particular, recent theoretical studies have shown that *Wolbachia*-induced CI can work in conjunction with geographically divergent selection on a nuclear locus to bring about reinforcement between populations [24]. Thus, *Wolbachia* may act as an agent for reinforcement of pre-existing isolating mechanisms, such as hybrid male sterility, and thus play a supplementary (not primary) role in the evolution of reproduction isolation between incipient species.

Although the most obvious consequence of reinforcement is decreased mating between individuals of different species, this process can also bring about behavioral isolation between sympatric and allopatric populations of the same species, as postulated theoretically [25] and as found in frogs [26,27] and *Drosophila* [28]. Thus, it is possible that secondary contact between a *Wolbachia*-infected species and a closely related uninfected species may bring about the evolution of behavioral isolation not only between the infected and uninfected species, but also between populations of the uninfected species.

We examined the evolutionary behavioral consequences of secondary contact between D. recens and D. subquinaria. Like most members of the quinaria species group, these two species are mycophagous, and mushrooms serve not only as larval food resources, but also as sites for adult courtship and mating. D. recens is infected with a strain of *Wolbachia* that causes strong intraspecific CI [29]. The prevalence of infection is about 98% among D. recens in the wild, which, along with dramatically reduced levels of mitochondrial DNA (mtDNA) diversity [13,30], indicates that the infection has spread to effective fixation. Reduced mtDNA variation is a common consequence of *Wolbachia* infection [31,32]. In contrast, the very closely related D. subquinaria is not infected with *Wolbachia*, and its high levels of mtDNA variation indicate that it has not been infected in the recent evolutionary past [13,32]. *Wolbachia*-induced CI causes a dramatic (~90%) reduction in offspring production in crosses between D. subquinaria females and D. recens males, but the reciprocal cross yields numerous *Wolbachia-*infected viable hybrid progeny [13]. The F1 hybrid progeny of the latter cross are viable because there is no CI. Although the hybrid males are sterile, the hybrid females are fertile and could thus serve as conduits for introgression of genes between species and for passing genes back into the parental species.

Previous work on this pair of species revealed only moderate behavioral isolation between them, suggesting that hybridization might frequently occur in the wild if these species were to come into contact. However, as pointed out by Rokas [33], the experimental strains of D. recens and D. subquinaria had been derived from allopatric populations, and thus might not have been selected to discriminate against males of the other species. Sympatric populations of these species, if they exist, might exhibit greater levels of discrimination. D. subquinaria is currently known from western North America, whereas *D. recens* is known only from eastern North America [34], although Shoemaker et al. [13] had speculated that the two may be sympatric in Canada, where there is a continuous zone of boreal forest (the preferred habitat of D. recens and D. subquinaria) that spans the region between the known ranges of these species.

We show here that the ranges of D. recens and D. subquinaria include a broad region of sympatry in central Canada. Within this zone of sympatry, D. subquinaria exhibits much greater reproductive character displacement than *D. recens,* which is consistent with the expected effects of *Wolbachia*-induced CI in hybrid offspring. In accordance with certain models of reinforcement [25,35], sympatric females of D. subquinaria not only discriminate against males of *D. recens,* but also against conspecific but allopatric males. Molecular data from mtDNA indicate that D. recens and D. subquinaria have hybridized in the past, as required by theories of reinforcement. Patterns of variation at the mitochondrial *Cytochrome Oxidase I (COI)* locus indicate that there is little genetic differentiation between sympatric and many allopatric populations within each species, suggesting that reproductive character displacement has evolved in the face of considerable gene flow and is likely the result of selection rather than genetic isolation among populations.

## Results

### Geographic Range

**Figure 1:**
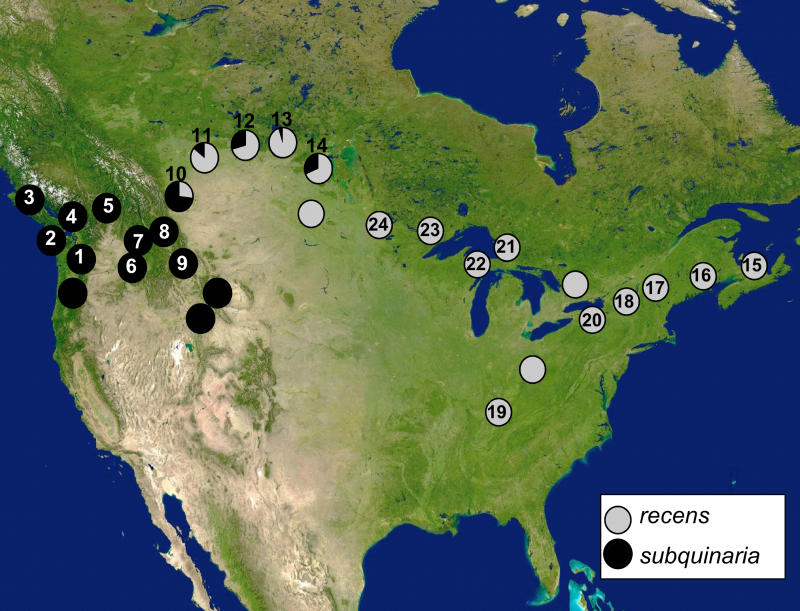
Collection Sites and Relative Abundance of D. recens (Gray) and D. subquinaria (Black) Satellite imagery courtesy of http://www.GlobeXplorer.com.

Our collections revealed a substantial area of sympatry between *D. recens* and *D. subquinaria,* extending, at a minimum, from Jasper, Alberta, to The Pas, Manitoba, a linear extent of some 1,200 km (Figure 1 and Table 1). Both species were collected simultaneously in this area of sympatry, indicating that they are likely to encounter each other at mushrooms in the field. Allopatric populations of *D. recens* occur throughout eastern Canada and the northeastern United States, and extend south through the Appalachians to high-elevation sites in the Smoky Mountains of Tennessee and North Carolina. Allopatric populations of D. subquinaria occur over much of western North America. Thus, there are sympatric and allopatric populations for both of these species.

**Table 1:**
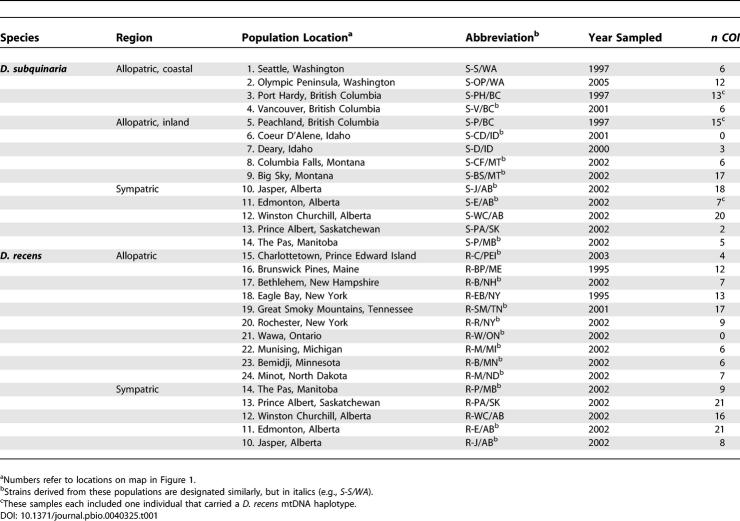
Populations and Strains Used in this Study and Number of Individuals from Each Population Sequenced at *COI*

### Mating Experiments—Mass Population

**Figure 2:**
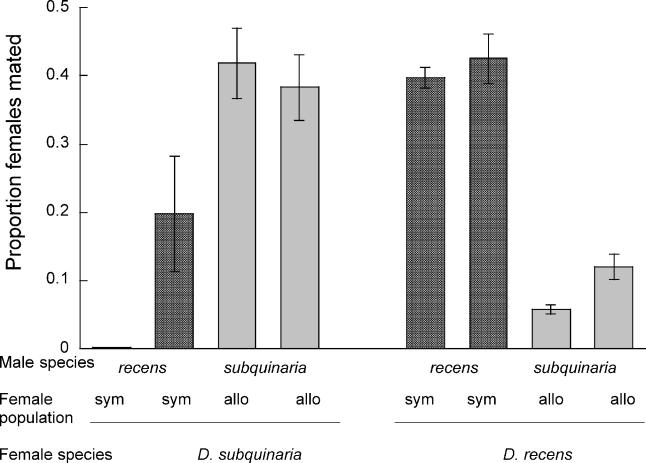
Proportion of Either Sympatric (sym) or Allopatric (allo) Females of D. recens and D. subquinaria That Mated with Either D. recens or D. subquinaria Males in the Mass Population Choice Experiments For example, within trials, an average of 20 of the 100 allopatric females of D. subquinaria mated with D. recens males and 38 of them mated with D. subquinaria. Means ± standard error among three replicate trials.

The proportion of sympatric and allopatric D. recens and *D. subquinaria* females that mated with either *D. recens* or *D. subquinaria* males is shown in Figure 2. In each of the three trials, the allopatric females of *D. subquinaria* were significantly more likely to mate with *D. recens* males than were sympatric females, with an across-trials combined probability of *p* = 10^−8^. In our experiments, sympatric females never mated with *D. recens* males. In contrast, the difference between sympatric and allopatric females of D. recens was nonsignificant within each trial, and the combined across-trials probability was *p* = 0.20. Thus, in the mass mating experiments, *D. subquinaria* females exhibited very strong reproductive character displacement, whereas *D. recens* females showed little.

### Mating Experiments—No Choice

**Figure 3:**
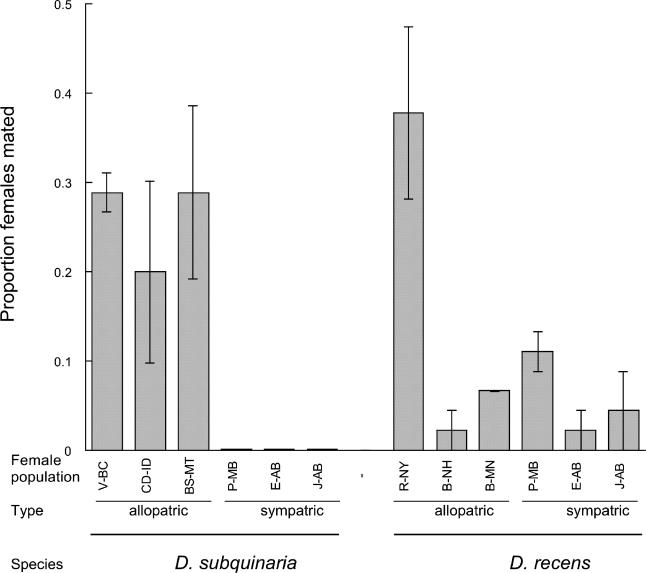
Proportion of Females That Mated with Heterospecific Males in the No-Choice Trials Means ± standard error among replicate trials. Females are organized by species, population type (sympatric or allopatric within species), and population within type. Population labels from Table 1, with S- or R- prefix deleted. The populations correspond with the sites in Figure 1 as follows: *V-BC,* 4; *CD-ID,* 6; *BS/MT,* 9; *P/MB,* 14; *E-AB,* 11; *J-AB,* 10; *R-NY,* 20; *B-NH,* 17; *B-MN,* 23.

Figure 3 presents the numbers of females from sympatric and allopatric populations of *D. recens* and *D. subquinaria* that mated with heterospecific males in no-choice trials. Although contextually very different from the mass population assays, the results are essentially the same. None of the sympatric females of *D. subquinaria* mated with *D. recens* males, whereas a considerable proportion (26% overall) of the allopatric females did. A logistic regression of the probability of mating by females of D. subquinaria revealed a highly significant effect of population type (sympatric versus allopatric; Wald χ^2^ = 17.88, *p* < 0.0001), but no effect of individual population within population type (χ^2^ = 1.02, *p* = 0.91) or trial (χ^2^ = 2.12, *p* = 0.35).

As with the mass mating experiment, we found little evidence for reinforcement within *D. recens*. A logistic regression of the probability of female mating revealed nonsignificant effects of population type (sympatric versus allopatric; Wald χ^2^ = 1.99, *p* = 0.16) and trial (χ^2^ = 0.50, *p* = 0.78). However, we did find a significant effect of individual population with population type (χ^2^ = 23.93, *p* = 0.0001), an effect due primarily to the much greater propensity of females of the allopatric *R-R/NY* strain to mate with *D. subquinaria* males (Figure 3).

### Intraspecific Mate Choice—No Choice

**Figure 4:**
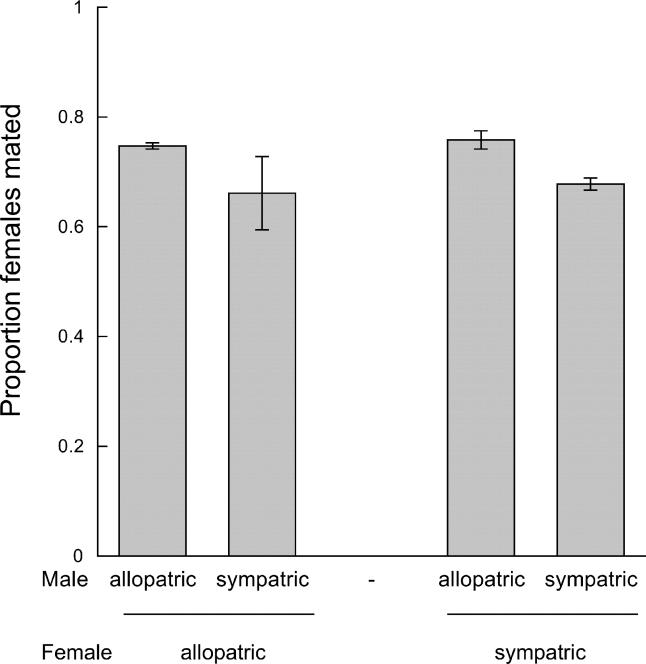
Proportion of D. recens Females from Either Sympatric or Allopatric Populations That Mated with Either Sympatric or Allopatric D. recens Males in No-Choice Tests Data from different allopatric populations combined and that from sympatric strains combined. Mean ± standard error among the two replicate trials.

We assessed whether there is any geographic differentiation in mate preference within each species. For *D. recens,* a logistic regression indicated that there was no significant effect of trial (Wald χ^2^ = 0.98, *p* = 0.32), female population type (sympatric versus allopatric; χ^2^ = 0.01, *p* = 0.93), male population type (χ^2^ = 3.62, *p* = 0.06), or female population type by male population type interaction (χ^2^ = 0.002, *p* = 0.96) on probability of a mating (Figure 4). Thus, there is no evidence of geographic differentiation in mate preferences.

**Figure 5:**
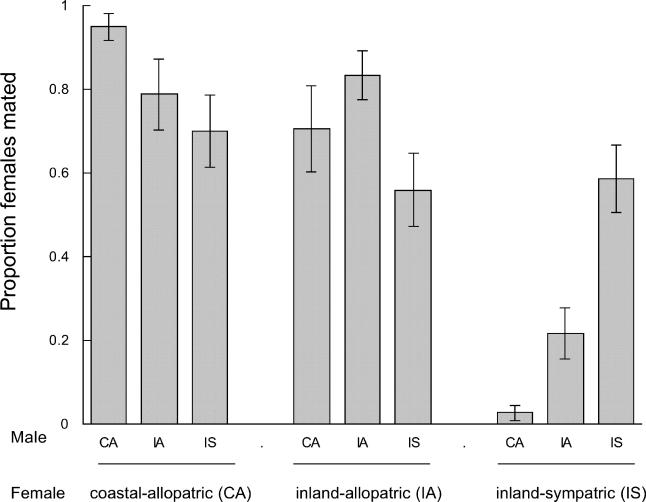
Proportion of D. subquinaria Females from Different Regions That Mated with D. subquinaria Males from These Same Regions in No-Choice Tests Coastal-allopatric (CA), inland-allopatric (IA), or inland-sympatric (IS). Mean ± standard error among the four replicate trials.

In *D. subquinaria,* there were highly significant effects of female population type (coastal-allopatric, inland-allopatric, and inland-sympatric; χ^2^ = 88.0, *p* < 0.0001) and female population type by male population type (χ^2^ = 62.6, *p* < 0.0001), and trial (χ^2^ = 12.3, *p* = 0.006), whereas the effect of male population type was not significant (χ^2^ = 1.80, *p* = 0.40). The significant effects were due to the low probability of mating between sympatric females and allopatric males, an effect that was consistent across trials. Because the significant female-by-male interaction could be due either to the coastal-inland differentiation or to the differences between inland-sympatric and inland-allopatric populations, we also analyzed the data including only the inland populations. In this case, we found significant effects of female population type (χ^2^ = 77.7, *p* < 0.0001), male population type (χ^2^ = 10.0, *p* = 0.0015), trial (χ^2^ = 11.6, *p* = 0.01), and, most importantly, female population type by male population type (χ^2^ = 62.4, *p* < 0.0001). Figure 5 illustrates that significant interaction effects are due to the reluctance of sympatric females to mate with males from both inland and coastal allopatric populations.

### Post-Mating Isolation within D. recens and D. subquinaria

To examine the extent of differentiation among conspecific populations of D. subquinaria and *D. recens,* we determined whether any qualitative post-mating isolating mechanisms operate between our strains. Within both species, all pairwise crosses among strains yielded viable male and female progeny. For each pairwise cross, all three replicate cultures that were set up as crosses between F1 male and F1 female progeny yielded viable male and female F2 progeny. Finally, for each pairwise mating, all three replicate cultures involving crosses between F2 males and females yielded viable male and female progeny. Thus, both male and female F1 and F2 are viable and fertile in all pairwise crosses between geographic strains within species. There is no evidence of hybrid sterility or inviability in any of these intraspecific crosses. Although sympatric females of *D. subquinaria* are reluctant to mate with allopatric males, prolonged confinement with allopatric males did yield matings and offspring. Thus, there is no evidence of qualitative post-mating isolation among any strains of *D. subquinaria,* and likewise for D. recens.

### Population Structure

**Figure 6:**
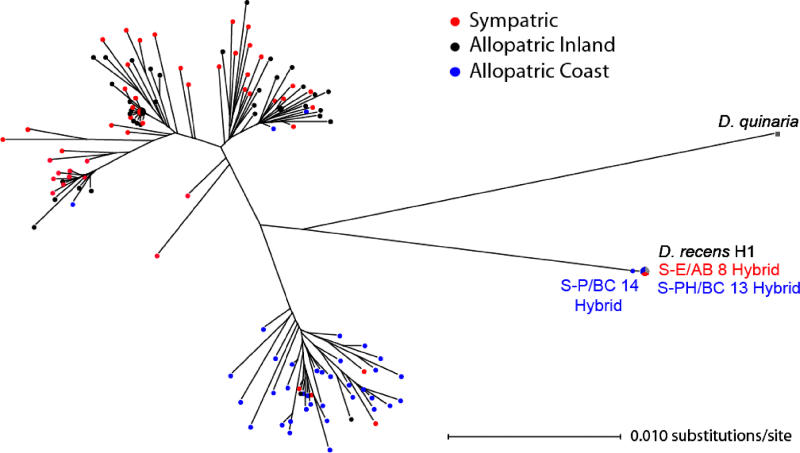
Minimum-Spanning Tree for *D. subquinaria COI* Red dots are samples collected from the inland-sympatric populations, black dots represent inland-allopatric samples, and blue represent coastal-allopatric populations. The sizes of the circles indicate number of samples with each haplotype. The individuals identified as hybrids carry mtDNA haplotypes typical of D. recens but nuclear genes from D. subquinaria. Note the difference in scale between this figure and that for D. recens (Figure 7).

**Figure 7:**
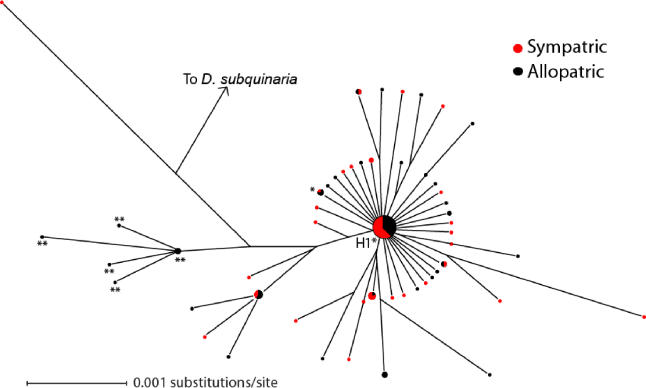
Minimum-Spanning Tree for *D. recens COI* Red dots are samples collected from the sympatric populations, and black dots are samples collected from the allopatric populations. Haplotypes marked with an asterisk were found in the Smokies as well as other areas, and those marked with double asterisks were found only in the Smokies. The areas of the circles are proportional to number of samples with each haplotype. H1 is the most abundant haplotype (*n* = 66). Distance to outgroups can be seen in Figure 6.

Both analyses of molecular variances and *F*_ST_-based statistics reveal that there is no overall significant differentiation between sympatric and allopatric groups of populations of either D. recens or *D. subquinaria,* although there is significant differentiation among populations within groups in both species (Table 2 and Protocol S1). Among the populations of *D. subquinaria,* there is significant differentiation between the coastal and inland populations (Figure 6 and Protocol S1). These regions are separated by the Pacific Cordillera and the high desert ecosystems of central Oregon, Washington, and British Columbia, suggesting that these areas constitute a substantial barrier to gene flow in *D. subquinaria,* as documented in other species of the Pacific Northwest [36]. Considering only the inland populations, which include all of the sympatric populations, there is no significant differentiation between those that are sympatric with D. recens and those that are allopatric (*S*_nn_ = 0.52; *p* = 0.69) (Figure 6). In D. recens, there is no significant differentiation between sympatric and allopatric populations (Table 2 and Figure 7), although there is significant within-group differentiation, due largely to the Great Smoky Mountains population, which occurs at the southern periphery of the species range (Figure 7). The Smokies population alone includes mtDNA haplotypes in both of the deepest branches of the mtDNA phylogeny, suggesting that the range of D. recens may have expanded out from this general region.

**Table 2:**
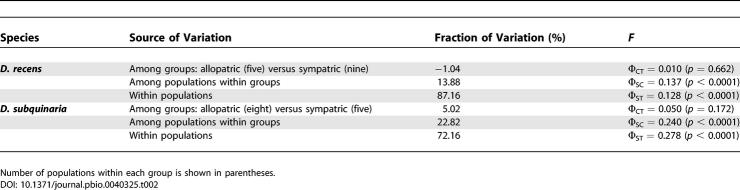
Molecular differentiation within and among Populations of D. recens and D. subquinaria.

Our samples included three individuals of D. subquinaria that carried mtDNA typical of D. recens and thus were of hybrid ancestry (Figure 6). These flies included two from allopatric populations—Port Hardy, British Columbia (S-PH/BC-13 [13]), and Peachland, British Columbia (S-P/BC-14)—and one from a sympatric population, Edmonton, Alberta (S-E/AB-8). To verify the hybrid origin of these individuals, each was sequenced at two nuclear loci, *R1B* and *period*. All three of these individuals carried D. subquinaria–specific sequences at both loci, and none was infected with *Wolbachia*. These three individuals were excluded from our population genetic analyses. No individuals of D. recens (identified by male genitalia and/or PCR assay for *Wolbachia* infection) carried a D. subquinaria mtDNA haplotype, and we found no evidence of heteroplasmy (as indicated by multiple peaks on the chromatogram).

## Discussion

Our field collections revealed a zone of geographical overlap between D. recens and D. subquinaria extending ~1,200 km from western Alberta to western Manitoba. Given the geological history of North America and the current ranges of these two species, it is likely that D. recens had been confined to eastern North America and D. subquinaria to western North America during the Wisconsin Glaciation and came into secondary contact when suitable habitat became connected across central Canada, i.e., within the last 12,000 y [37–39]. This zone of sympatry corresponds to Remington's suture zone 5 [40], which extends east from the eastern slope of the Canadian Rockies.

The principal aims of this study were to determine the following: (1) if sympatric populations of either D. recens or D. subquinaria exhibited reproductive character displacement; (2) if the species differed in the magnitude of this effect, in particular whether D. subquinaria not infected with *Wolbachia* exhibited greater reinforcement; (3) if there were any indirect effects of reinforcement on behavioral isolation within species; and (4) whether behavioral differentiation is simply an indirect consequence of general genetic differentiation among populations.

Females from sympatric populations of D. recens were somewhat (but not significantly) less willing to mate with D. subquinaria males than were females from allopatric populations in both the mass matings and the no-choice experiments. However, in the no-choice experiments, the higher level of heterospecific mating by allopatric females of D. recens was due entirely to one strain, *R-R/NY* (Figure 3). Thus, although there is evidently genetic variation within D. recens in willingness to mate with males of *D. subquinaria,* this variation is only weakly correlated with the sympatric or allopatric status of the D. recens populations. Thus, there is little evidence for reinforcement in D. recens.

In contrast, we found a very high level of reproductive character displacement in D. subquinaria: allopatric females mated quite readily with D. recens males, but the sympatric females consistently refused to do so. This dramatic difference between sympatric and allopatric females was seen in two very different behavioral assays: a mass population encounter, as might occur in the vicinity of attractive mushrooms in the wild, and a no-choice assay designed to assess whether females would mate with heterospecific males when no conspecific males were present. This finding is in contrast to the result of Coyne et al. [41], who showed that the degree of behavioral isolation between D. yakuba and D. santomea depended on the assay conditions, with isolation being significantly greater in choice assays. Our experiments show that the difference in mating behavior between sympatric and allopatric females of D. subquinaria is not context-dependent in the laboratory, suggesting that the difference is likely to be manifest under a variety of conditions in the wild.

To explore how *Wolbachia*-induced CI can lead to asymmetrical reinforcement between these two species, consider the following two possible initial crosses between them: (1) D. subquinaria female crossed with D. recens male and (2) D. recens female crossed with *D. subquinaria* male. For simplicity in what follows, assume that CI results in mortality of all offspring, although it is actually closer to 90% [13]. Cross 1 results in CI, causing an immediate loss of all alleles derived from both parental species. Cross 2, however, results in fertile hybrid females (hybrid males are sterile), which can mate with either D. recens or D. subquinaria males. Because these hybrids are infected with *Wolbachia,* backcross matings between hybrid females and D. recens males do not result in CI, thus allowing gene flow from the hybrids into D. recens. If hybrid females backcross to D. subquinaria males, the female descendants will be infected with *Wolbachia* and can continue to backcross to D. subquinaria males, because this is a compatible cross. However, the males produced in this backcross will be infected with *Wolbachia,* and their matings with D. subquinaria females (which are uninfected) will result in CI and thus the loss of genes that had been present in the original hybrids. In every backcross generation, half of the remaining nuclear genes that were present in the initial hybrids will segregate into males and thus be lost due to CI. Ultimately, all such nuclear genes will be lost in backcrosses to D. subquinaria.

The existence of unidirectional interspecific CI can also be inferred by patterns of mtDNA introgression between species. Because crosses between D. subquinaria females and D. recens males result in CI, introgression of D. subquinaria mtDNA into D. recens is greatly hindered. In contrast, crosses between D. recens females and D. subquinaria males do not entail CI, and by backcrossing of the hybrid female to *D. subquinaria,* the mtDNA of D. recens can introgress into D. subquinaria. Thus, asymmetric introgression of mtDNA between species is an indication of both interspecific hybridization and interspecific CI in the wild. We found that three of 130 D. subquinaria individuals carried a D. recens mtDNA haplotype, whereas no individuals of D. recens have been found with a D. subquinaria haplotype (*n* = 212 individuals, including results reported here and those in Shoemaker et al. [13,31]). The difference between D. recens and D. subquinaria in the proportion of flies carrying heterospecific mtDNA is of borderline significance (Fisher's exact test, two-tailed *p* = 0.054).

One effect of this unidirectional interspecific CI is that nuclear genes can introgress from D. subquinaria into *D. recens,* but not vice versa. The model of Telschow et al. [42] can be applied to this situation. The effective rate of nuclear gene flow from an uninfected *(D. subquinaria)* to an infected *(D. recens)* population is reduced by a fraction *L*/(1 + *L*), where *L* is the level of CI [42]. Thus, if *L* = 1 (100% offspring mortality), then gene flow is 50% less than it would be in the absence of CI, the reduction being due to cross 1 above between uninfected females *(D. subquinaria*) and infected males *(D. recens)*. In contrast, gene flow from the infected to the uninfected population is reduced by *L.* In other words, with complete CI (*L* = 1), there can be no introgression of genes from the infected into the uninfected species. Thus, the effect of CI is to allow some introgression of nuclear genes from D. subquinaria into *D. recens,* but none from D. recens into D. subquinaria.

How does unidirectional CI affect the evolution of mate choice? Suppose both species vary genetically in their level of discrimination against heterospecific males. Females of D. subquinaria that mate with D. recens males are likely to carry the less-discriminating alleles. However, because of CI, these alleles are transmitted to inviable progeny and are thereby selected out of the D. subquinaria population. In the reciprocal cross between D. recens females and D. subquinaria males, the less-discriminating alleles from D. recens are passed on to viable and fertile hybrid females. As discussed above, these alleles can then be crossed back into *D. recens,* but not into D. subquinaria. Thus, there is weaker selection against the less-discriminating alleles in D. recens than in *D. subquinaria,* the net effect being greater reinforcement in D. subquinaria.

There is an additional effect of the asymmetrical introgression discussed above. When D. subquinaria males and D. recens females mate and produce hybrid female offspring, these offspring are likely to carry alleles—which are transmitted by *D. subquinaria* males—that confer a female preference for D. subquinaria males. This is because the genes that affect mate choice by females may not have strong pleiotropic effects on the tendency of males to court and mate with various types of females, especially because males are the less-choosy sex in *Drosophila* [43]. Note that this is different from the effect of reduced female discrimination discussed above. Because genes can be introgressed from D. subquinaria to D. recens via these hybrid females, there can be introgression of *D. subquinaria–*preferring alleles into D. recens. Such introgression could potentially increase the tendency of sympatric D. recens females to mate with D. subquinaria males. In contrast, CI prevents the reciprocal introgression of *D. recens–*preferring alleles into D. subquinaria. Thus, D. subquinaria experiences greater selection against nondiscriminating alleles (reinforcement) and is less subject to introgression of alleles conferring a preference for heterospecific males. Therefore, the much greater level of reproductive character displacement observed in D. subquinaria is consistent with the postulated effects of unidirectional CI imposed by *Wolbachia* infection in D. recens.

An alternate explanation for the greater reproductive character displacement exhibited by D. subquinaria is that the sympatric populations of this species are less inundated by gene flow from allopatric populations than are those of D. recens. Thus, the behavioral difference between sympatric and allopatric populations of D. subquinaria might simply be a consequence of general genetic divergence among these populations. However, we found essentially no genetic differentiation between inland-sympatric and inland-allopatric populations of *D. subquinaria,* and females from the inland-allopatric populations from Big Sky, Montana (S-BS/MT), and Coeur D'Alene, Idaho (S-CD/ID), exhibit the same low level of discrimination against D. recens males as do females from the allopatric coastal populations (Figure 3). Thus, reproductive character displacement is apparent even between inland-sympatric and inland-allopatric populations, which exhibit little or no mtDNA differentiation. This pattern is consistent with the behavioral patterns being a direct result of natural selection, due to unidirectional CI, rather than a secondary consequence of general genetic differentiation.

The occurrence of D. subquinaria individuals carrying mtDNA haplotypes characteristic of D. recens indicates that these species were not completely reproductively isolated when they came into secondary contact, as required by models of reinforcement [3]. Three lines of evidence indicate that these introgressions are not recent events. First, two of the three individuals of D. subquinaria that carried D. recens haplotypes were collected in allopatric populations several hundred kilometers away from the zone of sympatry, a signature of past introgression [44]. Second, none of the three individuals was infected with *Wolbachia*. Loss of infection is expected to result from imperfect maternal transmission, acting over many generations, coupled with a lack of transmission advantage of CI-causing *Wolbachia* when the prevalence of infection is low [45,46]. Finally, one of these haplotypes is identical to the very common D. recens haplotype H1, differing only in a synonymous C-to-T substitution at site 7, which may have occurred after introgression of this haplotype into D. subquinaria.

Although we have focused on the role that *Wolbachia* may play in the evolution of reinforcement in *D. subquinaria,* an open question is why D. recens shows little reproductive character displacement. We suggest three possibilities. First, general theoretical models of reinforcement [47] show that there can be a threshold level of selection against hybrids above which reinforcement occurs and below which it does not. It is thus possible that the extra selective burden imposed by CI results in a surpassing of the threshold for D. subquinaria but not for D. recens. Second, as discussed above, sympatric populations of D. recens may be subject to some introgression of alleles that confer a female mating preference for D. subquinaria males, and such introgression would act to reduce reproductive character displacement. Finally, there may be some reproductive character displacement in D. recens, but our studies were not sensitive enough to detect it.

We found no post-mating isolation between the sympatric and allopatric populations of *D. subquinaria,* because both sexes of the F1 and F2 were viable and fertile in crosses between all pairwise combinations of populations. Although not tested yet, it is possible that more subtle forms of isolation operate between these populations, such as differential sperm precedence in multiply-mated females. In contrast, we did find substantial pre-mating behavioral isolation between D. subquinaria populations that are either sympatric or allopatric with D. recens. In our no-choice assays, we found that females of D. subquinaria from sympatric populations very often reject conspecific males from allopatric populations, both coastal and inland. In contrast, females from allopatric populations readily mate with conspecific males from both allopatric and sympatric populations. The finding that sympatric females discriminate against males from a genetically very similar inland-allopatric population suggests that reproductive character displacement in the sympatric populations, rather than general genetic differentiation, is responsible for the behavioral isolation among populations in D. subquinaria.

In general, behavioral isolation due to female mate choice can evolve as a result of changes in the mean male phenotype preferred by females or the range of male phenotypes against which females discriminate [25,48–50]. The pattern of behavioral isolation within D. subquinaria is not consistent with predictions of either a pure “mean preference” or pure “discrimination” model of behavioral isolation [25]. We propose the following simple model to explain the patterns of behavioral isolation observed in D. subquinaria. Suppose males of both D. recens and D. subquinaria display trait A, where A is a particular courtship song. As a result of selection to discriminate against males of *D. recens,* sympatric females of D. subquinaria accept only males that also display trait B, which may be a pheromone blend that distinguishes local (i.e., sympatric) D. subquinaria males from D. recens. Because there has been no selection for increased discrimination in allopatric populations, the display of male trait A is sufficient for acceptance by females of D. subquinaria from allopatric populations. If males from sympatric and allopatric populations of D. subquinaria differ in trait B, then these hypothetical mate preferences would yield the patterns of reinforcement and intraspecific behavioral isolation observed in D. subquinaria. This scenario is similar to the discrimination model of mate choice, because males displaying traits A and B are a subset of those displaying trait A.

Whatever the proximate cues may be, our data suggest that selection on D. subquinaria females to avoid D. recens males has indirectly brought about increased reproductive isolation between sympatric and allopatric populations of D. subquinaria. Because behavioral isolation is often the first step towards speciation in *Drosophila* [51], our results suggest that the presence of *Wolbachia* in one species may contribute to reproductive isolation within a different, uninfected species.

## Materials and Methods

### Geographical range and strain establishment.

We surveyed many *Drosophila* populations ranging from British Columbia in the west to Prince Edward Island in the east, including a number of sites where D. recens and D. subquinaria were hypothesized to be sympatric [13]. Flies were collected by sweep netting over mushrooms from 1995 to 2005, with most collections in 2002 (Table 1). Males were dissected and identified to species by examining their genitalia, which clearly differ between these two species [25]. Females of D. recens or D. subquinaria were placed in culture to examine their sons for species identification and to establish strains for the mating experiments. Following dissection (males) or culture (female), wild-caught flies were frozen for subsequent population genetic analysis.

### Mating experiments—mass population.

Genetically heterogeneous strains of each species were created by combining virgin males and females from several isofemale lines that originated from multiple locations within the allopatric and sympatric region. The sympatric populations for both *D. recens* and *D. subquinaria* included strains from Jasper, Edmonton, and The Pas *(R-J/AB, R/E-AB, R-P/MB, S-J/AB, S-E/AB,* and *S-P/MB);* the allopatric population of *D. subquinaria* included strains *S-V/BC, S-CF/MT, S-BS/MT,* and *S-CD/ID;* and the allopatric population for *D. recens* included strains *R-M/ND, R-B/MN, R-M/MI, R-W/ON, R-R/NY, R-B/NH, R-C/PEI,* and *R-SM/TN*.

The experimental flies were reared on Formula 4–24 instant *Drosophila* medium (Carolina Biological Supply, Burlington, North Carolina, United States) plus commercial Agaricus bisporus mushroom. Stocks were set up with a consistent starting density of adult flies to ensure a similar rearing density of larvae. Upon emergence as adults, flies were isolated on light CO_2_ and kept as virgins on the same food at 22 °C with a 12-h light/dark cycle. The mating experiments were conducted when the males and females were 7 d post-emergence and only with flies with undamaged wings. All behavioral trials were conducted by aspirating flies into mating chambers, without the use of CO_2_.

Three mass population trials were carried out, each using a different generation of flies. Each trial included two population cages run simultaneously, one to assess the mating preference of *D. recens* females and one for *D. subquinaria* females. Each trial began by transferring virgin females without anesthesia 1 h after lights-on to a population cage, which contained mushrooms placed on top of moistened paper towel. The mushrooms were intended to provide a rendezvous site for courting flies and mimicked natural situations where scores of flies can be found on and around a single attractive mushroom in the wild.

In the first cage, the females included 100 D. subquinaria from allopatric populations and 100 from sympatric populations. In a second cage, the females included 100 D. recens from allopatric populations and 100 from sympatric populations. To distinguish the sympatric from allopatric females, one type was marked lightly with fluorescent dust. Sympatric females were marked in trials 1 and 3, whereas the allopatric females were marked in trial 2. Males were added to each cage about 5 min after the females were, also without using anesthesia. Each cage received 100 *D. subquinaria* and 100 *D. recens* males, which included both sympatric and allopatric populations. Males were marked with fluorescent dust to distinguish the species, but not whether they were from allopatric or sympatric populations; D. subquinaria males were marked in trials 1 and 3, whereas *D. recens* males were marked in trial 2.

After addition of the males, the cages were observed continuously. Copulating pairs were removed by aspiration and immediately examined under ultraviolet light to identify them. We collected the first 100 mating pairs, or all mating pairs that mated in the first 2 h, whichever came first. All remaining flies were subsequently aspirated from the cage and identified under ultraviolet light.

### Mating experiments—no choice.

In contrast to the mass population experiment, a no-choice design allows the determination of whether females of one species will mate with males of another species when deprived of an opportunity to mate with their own species. Flies were reared exactly as in the mass population experiment. In the no-choice experiment, females from a genetically mixed strain from a single population were placed individually with males of the other species. For each species, we tested females from three allopatric populations and three sympatric populations. The allopatric populations of D. subquinaria used were strains *S-V/BC, S-CD/ID,* and *S-BS/-MT;* the sympatric populations for both species were the same as those used in the mass population experiment; and the allopatric populations of D. recens included strains *R-R/NY, R-B/MN,* and *R-B/NH. D. subquinaria* females were paired with *D. recens R/E-AB* males, and each D. recens strain was paired with equal numbers of *D. subquinaria S-CD/ID, S-E/AB,* and *S-J/AB* males.

To test female mating behavior in a no-choice situation, one each of a 7-d-old female and male and were aspirated into a small vial (1 cm diameter × 3 cm long) containing blended mushroom-agar medium. The pairs consisted of either an allopatric or sympatric female of one species paired with a male of the other species. Three separate trials were conducted, and in each trial, we tested five females from each of three allopatric and three sympatric populations of each species. The number of females that mated within the first 3 h was counted.

### Intraspecific mate choice—no choice.

To assess whether there is any geographic differentiation in mate preference within species, we used the same methods as for the no-choice experiment, but paired males and females from the same species. For *D. recens,* we used flies from three sympatric *(R-E/AB, R-J/AB,* and *R-P/MB)* and three allopatric populations *(R-B/NH, R-B/MN,* and *R-R/NY)* in all pairwise combinations in two independent trials. For *D. subquinaria,* in the first two trials we used flies from two inland-sympatric *(S-E/AB* and *S-J/AB),* two inland-allopatric *(S-CD/ID* and *S-BS/MT),* and one coastal-allopatric population *(S-V/BC)*. Two additional trials were carried out using three strains of D. subquinaria: combined *S-E/AB* and *S-J/AB* = *S-JE/AB* (inland-sympatric), *S-CD/ID* (inland-allopatric), and *S-V/BC* (coastal-allopatric). The use of D. subquinaria from these populations enabled us to assess whether intraspecific discrimination was associated with the inland-coastal genetic structure of D. subquinaria or the allopatric-sympatric distinction among genetically similar populations. The proportion of females that mated was analyzed via logistic regression as a function of trial, female population type, male population type, and interaction between the female and male population types.

### Post-mating isolation within D. recens and *D. subquinaria.*

Because we found evidence of behavioral isolation within *D. subquinaria,* we wished to determine whether taxonomically conspecific individuals from different populations exhibit post-mating isolation. Using the same genetically variable strains as for the intraspecific mate choice experiment, we did crosses of all reciprocal pairwise combinations (30 combinations within D. recens and 20 within D. subquinaria), each starting with three males and three females. For each, we then set up three crosses using the F1 offspring and, subsequently, three crosses using the F2 offspring, where each cross included three males and three females. For each of the F1 and F2 crosses, we scored whether the flies produced viable offspring, and in this manner, we were able to assess the viability and fertility of F1 and F2 offspring produced by inter-population crosses.

### Population structure.

To assay differentiation among populations as well as the frequency of hybrids, we sequenced 1,432 base pairs of the mtDNA *Cytochrome Oxidase I (COI)* gene. Although the mtDNA of D. recens is known to harbor low polymorphism [31], there is no evidence that *COI* is currently experiencing a selective sweep, and thus the present polymorphism should reflect recent patterns of gene flow among populations. We sequenced *COI* from wild-caught individuals or single flies from isofemale lines established from wild flies of D. subquinaria and D. recens. Some of our samples were obtained from isofemale lines established from wild-caught females. In these cases, species identifications were made by dissection and examination of male genitalia, which show diagnostic differences between D. recens and D. subquinaria [35]. Some wild-caught individuals were frozen before dissection; to provisionally identify these flies, we conducted a PCR assay to test for *Wolbachia* infection (using the primers wsp81F and wsp691R from Zhou et al. [52]). Individuals that tested positive for *Wolbachia* were tentatively identified as *D. recens,* and uninfected flies as D. subquinaria. Because about 2% of wild-caught D. recens are uninfected due to imperfect maternal transmission of *Wolbachia* [13], the few uninfected flies that had a *D. recens COI* haplotype were also sequenced at a nuclear marker (as in [31]) to determine whether they were uninfected D. recens or a D. subquinaria that carried a *D. recens COI* haplotype and thus of hybrid origin. Samples which carried a *D. recens–*type *COI* but a *D. subquinaria–*type nuclear marker were sequenced at a second nuclear marker to confirm species identity. Nuclear markers used were *period* and *R1B*. In all three of these cases, both nuclear markers were of D. subquinaria type but the individual carried a D. recens type mtDNA, indicative of a historical introgression of the mtDNA.

We used standard techniques for all PCR and DNA sequencing (Protocol S1). Sequences are deposited in GenBank and were combined with previously published sequences.

Within each species, we tested for differentiation between sympatric and allopatric regions and for differentiation between all pairs of populations. We used variants of *F*_ST_ statistics, as implemented in DnaSP 4.0 [53] (Protocol S1). In addition, we partitioned variation within and among sympatric and allopatric regions using an analysis of molecular variance, as implemented in Arlequin [54].

To visualize relationships and relative abundances of mtDNA haplotypes within each species, a network was created in SplitsTree4 [55] (available from http://www.splitstree.org). A network was created for D. recens and D. subquinaria separately, using D. quinaria and one sample the other species as outgroups. For the networks shown in Figures 6 and 7, we used a neighbor-joining algorithm and Kimura 2-parameter distance estimates.

## Supporting Information

Protocol S1 Population Structure: Methods and Additional Results (133 KB DOC)

### Accession Numbers

The GenBank (http://www.ncbi.nlm.nih.gov/Genbank) accession numbers for sequences discussed in the paper are nuclear markers *period* and *R1B* in *D. recens* (AF248076 and DQ494185–DQ494192), nuclear markers *period* and *R1B* in *D. subquinaria* (DQ494178–DQ494184), and mtDNA *Cytochrome Oxidase I (COI)* from *D. recens, D. subquinaria,* and D. quinaria (AY154400–AY154457 and DQ851591–DQ851807).

## Abbreviations

CI: cytoplasmic incompatibility
mtDNA: mitochondrial DNA
