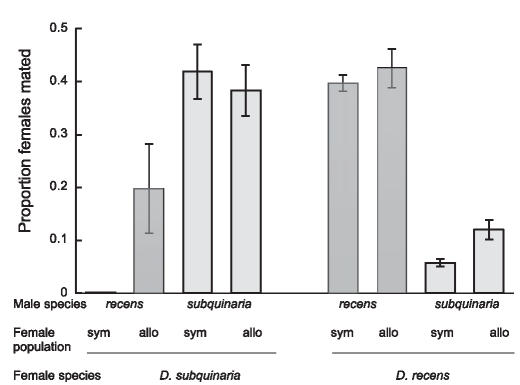# Correction: Asymmetrical Reinforcement and Wolbachia Infection in Drosophila

**DOI:** 10.1371/journal.pbio.0050003

**Published:** 2007-01-16

**Authors:** John Jaenike, Kelly Dyer, Chad Cornish, Miranda Minhas

In *PLoS Biology*, volume 4, issue 10: doi:10.1371/journal.pbio.0040325

The “female population” part of the x-axis label in Figure 2 has an error: the labels sym and allo are switched in two places. Attached is a PDF of Figure 2 as it should appear. The legend should remain the same.

## 

**Figure:**