# Supplementary material for: Asymmetrical Reinforcement and Wolbachia Infection in Drosophila
Source: PLoS Biol. 2006 Oct 10;4(10):e325. doi: 10.1371/journal.pbio.0040325 (PMC1592313; doi:10.1371/journal.pbio.0040325)
Supplement: Supplementary file 1 [file pbio.0040325.sd001.doc]

Jaenike et al: Asymmetrical Reinforcement and *Wolbachia* Infection n *Drosophila*

# Supporting Information – Population Structure

### *Methods*

###	To assay differentiation among populations as well as the frequency of hybrids, we sequenced 1432 bp of the *Cytochrome Oxidase I* (*COI*) gene from wild-caught sympatric and allopatric individuals of *D. subquinaria* and *D. recens*.

### We used standard techniques for all PCR and DNA sequencing. DNA was extracted from single flies using the Gentra Puregene kit (Gentra Systems, Minneapolis MN), *COI* was amplified using the primers TL-2-1460 and TY-N-3014 from [1], PCR amplicons were purified using Exosap-IT (United States Biochemical, Cleveland OH) and then directly sequenced using Big Dye Terminator chemistry (Applied Biosystems, Foster City, CA), base calls were verified using Sequencher (Gene Codes, Ann Arbor MI), and sequences were aligned manually using Se al [2].

Levels of intra-population genetic variability were quantified using the program DnaSP 4.0 [3]. Within each species, we tested for differentiation between sympatric and allopatric regions and for differentiation between all pairs of populations, as described in the main text. In addition, we used the permutation-based statistics *H*_ST_ [4], which is an estimator of Nei’s *G*_ST_ [5] and is a haplotype frequency-based statistic appropriate for *COI* in *D. recens* (which has very low *COI* polymorphism), and *S*_nn_ [6], which is a DNA sequence-based statistic that is more powerful for high levels of polymorphism as found in *D. subquinaria* [see 4,6]. We also tested for a correlation between geographic and genetic distance among populations using a Mantel test [7], as implemented in the program Isolation by Distance v2.6 [8]. For the analyses we used the natural logarithms of geographic distances [9] and pairwise *F*_ST_ with the adjustment of *F*_ST_/(1 - *F*_ST_) for genetic distance [10], with significance determined by 10000 matrix randomizations.

To visualize relationships and relative abundances of mtDNA haplotypes within each species, a median-joining network was created in SplitsTree4 [11]. To infer support values, we utilized MrBayes v 3.1 [12]. Under the HKY85 model with Γ-distributed rate variation, three separate runs were performed, each of which ran for 5 million generations and was sampled every 100 generations. The first 500,000 generations were excluded as burn-in. The final most credible tree was a 50% majority rule tree based on the subsequent 45,000 sampled trees, where the number of times a bipartition is recovered is directly proportional to the probability of support for that branch.

###### Results

The *COI* data set included 130 *D. subquinaria* (78 allopatric and 52 sympatric) and 144 *D. recens* (81 allopatric and 63 sympatric); sample sizes from each population are listed in Table 1 of the main text.

***D. recens***. Over all 144 *D. recens* samples, the 1432 bp fragment of *COI* had 58 variable sites, of which 37 were singletons and 21 were parsimony informative, and which comprised 53 unique haplotypes. This results in a species-wide pairwise nucleotide diversity of 0.00134 (0.00561 at silent sites), with individual populations ranging from 0.00024 to 0.00319 (Table S1). There is a significant amount of differentiation among populations, with *H*_ST_ = 0.0589 (*P* = 0.001) and *S*_nn_ = 0.158 (*P* < 0.0001). We found no evidence of isolation by distance among *D. recens* populations (*r* = 0.128, *P* = 0.10).

Differentiation between each pair of populations is shown in Table S1. There is no difference in the number of significantly different pairs within regions (allopatric *vs*. allopatric and sympatric *vs*. sympatric) compared to between regions (allopatric *vs*. sympatric) (significant pairs: *H*_ST_: 5/56 within region vs. 3/45 between region; *S*_nn_: 17/56 within and 10/45 between region). As Table S1 shows, R-C/PEI and R-SM/TN are differentiated from most of the other populations. These populations occur on the periphery of the range of *D. recens* (R-SM/TN at the southern and R-C/PEI at the northeastern edge).

When *D. recens* populations were grouped into those that are allopatric and sympatric with *D. subquinaria*, an AMOVA showed that regional variation (i.e., sympatric versus allopatric) explained essentially none of the total molecular variance (Φ_CT_ = 0.010, P = 0.662; Table S3), but differences among populations within regions accounted for 13.0% of the variation (Φ_SC_ = 0.137, *P* < 0.0001). In contrast, when populations were divided into two different groups – R-SM/TN versus all others – regional variation explained 32.8% of the total molecular variance (Φ_CT_ = 0.327, *P* = 0.072) and much less of the within-group variation (2.4%, Φ_SC_ = 0.036, *P* < 0.0001). Thus, the significant structure in *D. recens* is due largely to the R-SM/TN and, to a lesser extent, the R-C/PEI populations, both of which are allopatric to *D. subquinaria*.

***D. subquinaria***. Over all the 129 *D. subquinaria* samples, the 1432 bp fragment of *COI* had 187 variable sites, of which 77 were singletons and 110 were parsimony informative, and which comprised 121 unique haplotypes. This results in a species-wide pairwise nucleotide diversity of 0.01497 (0.0639 at silent sites), with individual populations ranging from 0.006 to 0.017 (Table S2). While we present *H*_ST_ for comparison with *D. recens*, we will only consider results from *S*_nn_ for *D. subquinaria COI*, as this is a more relevant statistic given the higher level of polymorphism. Among all *D. subquinaria* populations, there is a significant amount of differentiation among populations, with *S*_nn_ = 0.203 (*P* < 0.0001). In addition, there is a weak effect of isolation by distance (*r* = 0.25, *P* = 0.039).

Differentiation between each pair of populations is shown in Table S2. The proportion of significant differences among populations in all pairwise sympatric-allopatric comparisons (16/40) was similar to the proportion of significant differences within regions, i.e., sympatric-sympatric or allopatric-allopatric (13/38). All of the significant pairwise differences are between the coastal populations (S-OP/WA, S-S/WA, S-V/BC, and S-PH/BC) populations and the nine remaining inland populations, all of which lie to the east (Table S2).

The patterns observed in Table S2 are supported in the AMOVA analyses (Table S3). When populations are divided into the two regions that are allopatric or sympatric with *D. recens*, regional variation explained 5% of the total molecular variance (Φ_CT_ = 0.050, P = 0.17; Table S3), but among populations within regions explained 22.3% of the variation (Φ_SC_ = 0.240, *P* < 0.0001). In contrast, if populations are divided into coastal versus inland regions, 44.3% of the molecular variance is explained by regional differences (Φ_SC_ = 0.437, *P* < 0.0001) and less than 1% by variation among populations within regions (Φ_SC_ = 0.006, *P* < 0.0001). Finally, if we consider three groups of populations – coastal, inland allopatric, and sympatric – regional differences account for 31% of the variation, which is significant (Φ_CT_ = 0.319, *P* < 0.0001), but less so than the model that includes only coastal and inland regions. These observations are supported by the phylogenetic analysis of *COI* in *D. subquinaria*. Separation of the inland and coastal clades (as seen in Figure 7 of the main text) was supported in all of the sampled trees, whereas the relatively few of the nodes within each clade with high support values were on the tips of the tree (data not shown).

It is relevant to ask, then, whether there is significant population structure between the inland populations that are allopatric (S-P/BC, S-CD/ID, S-CF/MT, S-BS/MT) and those that are sympatric (S-E/AB, S-J/AB, S-WC/AB, S-PA/SK, S-P/MB) with *D. recens*. Between these regions, *S*_nn_ = 0.524 (*P* = 0.691), with several other standard differentiation statistics also showing no structure between these populations (data not shown). In addition, an AMOVA of this grouping of populations shows that ~100% of the molecular variation is present within populations and none of the variance comes from among regions or among populations within regions (Table S3).

In summary, the analyses of population differentiation of *D. subquinaria COI* consistently depicts major structuring of populations, but with the major divide between the coastal and inland populations. We find no evidence of decreased gene flow between sympatric and allopatric inland populations of either *D. subquinaria*.

**References**

1. Simon C, Frati F, Beckenbach A, Crespi B, Liu H, Flook P (1994) Evolution, weighting, and phylogenetic utility of mitochondrial gene sequences and a compilation of conserved polymerase chain reaction primers. Ann Entomol Soc Am 81: 651-701.

2. Rambaut A. (1996) Se-Al: Sequence Alignment Editor. <http://evolve.zoo.ox.ac.uk/>.

3. Rozas J, Sanchez-DelBarrio JC, Messeguer X, Rozas R (2003) DnaSP, DNA polymorphism analyses by the coalescent and other methods. Bioinformatics 19: 2496-2497.

4. Hudson RR, Boos DD, Kaplan NL (1992). A statistical test for detecting geographic subdivision. Mol Biol Evol 9: 138-151.

5. Nei M (1973) Analysis of gene diversity in subdivided populations. Proc Natl Acad Sci USA 70: 3321-3323.

6. Hudson RR (2000) A new statistic for detecting genetic differentiation. Genetics 155: 2011-2014.

7. Mantel N (1967) The detection of disease clustering and a generalized regression approach. Cancer Res 27: 209-220.

8. Jensen JL, Bohonak AJ, Kelley ST (2005) Isolation by distance, web service. BMC Genetics 6: 13. http://phage.sdsu.edu/~jensen/

9. Slatkin M (1993) [Isolation by distance in equilibrium and nonequilibrium populations](http://wos.isiknowledge.com:80/?SID=E254EFJg8AI@18gbjle&Func=Abstract&doc=1/4). Evolution 47: 264-279.

10. Rousset F (1997) [Genetic differentiation and estimation of gene flow from F-statistics under isolation by distance](http://wos.isiknowledge.com:80/?SID=A5pdoFBNMdhi5c8bGnD&Func=Abstract&doc=1/6). Genetics 145: 1219-1228.

11. Huson DH, Bryant D (2006) Application of phylogenetic networks in evolutionary studies. Mol Biol Evol 23:254-267. Software available from [www.splitstree.org](http://www.splitstree.org/).

12. Ronquist F, Huelsenbeck JP (2003) [MrBayes 3: Bayesian phylogenetic inference under mixed models](http://wos.isiknowledge.com:80/?SID=A5pdoFBNMdhi5c8bGnD&Func=Abstract&doc=11/5). Bioinformatics 19: 1572-1574.

**Table S1.** Polymorphism within and pairwise differentiation among *D. recens* populations at *COI*. The diagonal is the within population pairwise nucleotide diversity (π,x100). *H*_ST_ is shown below the diagonal and *S*_nn_ above diagonal, with *P*-values in parenthesis and pairs which are significant at the 0.01 level in bold). Overall, *D. recens* *COI* has π = 0.00134, and populations show significant differentiation, with *H*_ST_ = 0.0589 (*P* = 0.001) and *S*_nn_ = 0.158 (*P* < 0.0001). Populations are listed by their approximate location east to west, with the bold lines separating populations that are allopatric and sympatric with *D. subquinaria*.

Table S1 cont’d

|  |  |  |  |  | Allopatric | |  |  |  |  |  |  | Sympatric | |  |  |
| --- | --- | --- | --- | --- | --- | --- | --- | --- | --- | --- | --- | --- | --- | --- | --- | --- |
|  | C/PEI | BP/ME | B/NH | EB/NY | | SM/TN | R/NY | M/MI | B/MN | M/ND | PA/SK | | P/MB | WC/AB | J/AB | E/AB |
| C/PEI | *0.319* | **0.875**  **(0.001)** | **0.818**  **(0.006)** | **0.882**  **(0.000)** | | **0.904**  **(0.001)** | **0.846**  **(0.010)** | 0.700  (0.022) | **0.800**  **(0.000)** | **0.818**  **0.000)** | 0.769  (0.018) | | 0.769  (0.014) | **0.900**  **(0.002)** | 0.761  (0.017) | **0.916**  **(0.001)** |
| BP/ME | **0.184**  **(0.007)** | *0.047* | **0.613**  **(0.016)** | 0.480  (0.304) | | **0.730**  **(0.001)** | 0.617  (0.030) | **0.617**  **(0.003)** | 0.510  (0.580) | 0.518  0.189) | 0.478  (0.495) | | 0.460  (0.705) | 0.481  (0.580) | **0.744**  **(0.000)** | 0.468  (0.960) |
| B/NH | 0.102  (0.060) | 0.028  (0.183) | *0.128* | 0.629  (0.024) | | **0.768**  **(0.007)** | 0.456  (0.511) | 0.423  (0.358) | 0.545  (0.158) | 0.542  0.133) | 0.471  (0.430) | | 0.463  (0.343) | 0.541  (0.620) | 0.472  (0.366) | 0.692  (0.040) |
| EB/NY | **0.197**  **(0.003)** | -0.018  (0.070) | 0.037  (0.097) | *0.065* | | **0.736**  **(0.000)** | 0.563  (0.172) | **0.636**  **(0.004)** | 0.526  (0.520) | 0.534  .0156) | 0.565  (0.013) | | 0.498  (0.268) | 0.515  (0.359) | **0.762**  **(0.000)** | 0.479  (0.744) |
| SM/TN | 0.059  (0.020) | **0.081**  **(0.004)** | 0.036  (0.103) | 0.092  (0.003) | | *0.173* | **0.788**  **(0.000)** | 0.678  (0.075) | **0.739**  **(0.010)** | **0.726**  **0.007)** | **0.725**  **(0.004)** | | **0.728**  **(0.000)** | **0.692**  **(0.000)** | **0.765**  **(0.003)** | **0.732**  **(0.000)** |
| R/NY | 0.063  (0.069) | 0.039  (0.125) | -0.037  (1.000) | 0.017  (0.213) | | 0.033  (0.051) | *0.118* | 0.481  (0.421) | 0.611  (0.067) | 0.603  0.049) | 0.516  (0.227) | | 0.508  (0.201) | 0.560  (0.158) | 0.529  (0.207) | 0.662  (0.055) |
| M/MI | 0.034  (0.132) | 0.089  (0.050) | -0.011  (0.623) | 0.102  (0.540) | | 0.019  (0.191) | -0.016  (0.685) | *0.165* | 0.472  (0.000) | 0.467  (0.442) | 0.473  (0.544) | | 0.480  (0.514) | 0.580  (0.484) | 0.423  (0.648) | 0.727  (0.046) |
| B/MN | 0.338  (0.013) | -0.033  (1.000) | 0.048  (0.210) | -0.034  (1.000) | | 0.078  (0.026) | 0.051  (0.172) | 0.137  (0.182) | *0.024* | 0.452  0.662) | 0.490  (0.514) | | 0.478  (0.566) | 0.564  (0.518) | 0.642  (0.014) | 0.600  (0.743) |
| M/ND | 0.143  (0.030) | -0.012  (0.669) | -0.004  (0.575) | -0.007  (0.654) | | 0.040  (0.078) | 0.001  (0.350) | 0.035  (0.289) | -0.012  (0.549) | *0.061* | 0.500  (0.239) | | 0.483  (0.268) | 0.573  (0.327) | **0.667**  **(0.005)** | 0.619  (0.388) |
| PA/SK | 0.053  (0.061) | 0.023  (0.207) | -0.026  (0.842) | 0.040  (0.126) | | 0.026  (0.109) | -0.023  (1.000) | -0.014)  (0.796 | 0.039  (0.114) | -0.008  (0.657) | *0.181* | | 0.459  (0.506) | 0.479  (0.750) | 0.547  (0.222) | 0.542  (0.657) |
| P/MB | 0.196  (0.013) | -0.021  (1.000) | -0.003  (0.439) | -0.019  (1.000) | | 0.068  (0.019) | 0.012  (0.506) | 0.070)  (0.106) | 0.034  (1.000) | -0.020  (0.685) | 0.011  (0.342) | | 0*.*075 | 0.475  (0.921) | 0.551  (0.161) | 0.561  (0.465) |
| WC/AB | 0.088  (0.017) | -0.007  (0.615) | -0.017  (0.643) | 0.001  (0.376) | | 0.047  (0.019) | -0.006  (0.570) | 0.018  (0.223) | -0.008  (0.625) | -0.016  (0.854) | -0.009  (0.655) | | -0.019)  (0.820) | *0.086* | 0.655  (0.031) | 0.508  (0.301) |
| J/AB | 0.027  (0.082) | **0.138**  **(0.004)** | 0.031  (0.072) | **0.154**  **(0.001)** | | 0.046  (0.012) | 0016  (0.366) | -0.011  (1.000) | 0.176  (0.005) | 0.077  (0.029) | 0.016  (0.207) | | **0.121**  **(0.009)** | 0.064  (0.022) | *0.294* | **0.827**  **(0.000)** |
| E/AB | **0.068**  **(0.007)** | -0.008  (0.600) | 0.002  (0.290) | -0.006  (0.570) | | 0.047  (0.014) | 0.005  (0.299) | 0.025  (0.121) | -0.007  (0.552) | -0.013  (0.895) | -0.007  (0.637) | | -0.008  (0.632) | -0.009  (0.775) | **0.060**  **(0.009)** | *0.087* |

**Table S2.** Polymorphism within and pairwise differentiation among *D. subquinaria* populations at *COI*. The diagonal is the within population pairwise nucleotide diversity (π,x100). *H*_ST_ is shown below the diagonal and *S*_nn_ above diagonal, with *P*-values in parenthesis and pairs which are significant at the 0.01 level in bold. Overall, *D. subquinaria* has π = 0.01497, and populations show significant differentiation, with *H*_ST_ = 0.001 (*P* = 0.243) and *S*_nn_ = 0.203 (*P* < 0.0001). Populations are listed by their approximate location west to east; the bold black lines separate populations that are allopatric and sympatric with *D. recens*. The bold red lines separate populations to the west and east of the Coastal mountain range.

Table S2 cont.

|  |  |  | |  |  | Allopatric | | | | |  |  | |  |  |  | Sympatric |  | | | | |  |
| --- | --- | --- | --- | --- | --- | --- | --- | --- | --- | --- | --- | --- | --- | --- | --- | --- | --- | --- | --- | --- | --- | --- | --- |
|  |  | Coastal | | | |  |  |  |  |  | Inland | | | | | | |  |  |  | |  |  |
|  | OP/WA | | S/WA | V/BC^1^ | | PH/BC | | P/BC | | | D/ID | CF/MT | BS/MT | | E/AB | J/AB | WC/AB | P/MB | | | PA/SK | | |
| OP/WA | *1.440* | | 0.508  0.583 | 0.395  0.870 | | 0.506  0.371 | | 0.722  0.014 | | | **0.875**  **0.004** | 0.815  0.011 | 0.666  0.033 | | 0.772  0.031 | 0.703  0.019 | 0.487  0.597 | **0.890**  **0.000** | | | 0.656  0.592 | | |
| S/WA | 0.000  1.000 | | *0.642* | 0.750  0.035 | | 0.653  0.135 | | **0.950**  **0.000** | | | **1.000**  **0.000** | **1.000**  **0.000** | **0.935**  **0.001** | | **1.000**  **0.000** | **0.958**  **0.000** | **0.942**  **0.000** | **1.000**  **0.000** | | | **0.889**  **0.000** | | |
| V/BC^1^ | 0.000  1.000 | | 0.000  1.000 | *0.754* | | 0.574  0.332 | | **0.950**  **0.001** | | | **1.000**  **0.000** | **1.000**  **0.000** | **0.934**  **0.001** | | **1.000**  **0.000** | **0.958**  **0.000** | **0.852**  **0.006** | **1.000**  **0.000** | | | **0.889**  **0.009** | | |
| PH/BC | 0.000  1.000 | | 0.000  1.000 | 0.000  1.000 | | *0.724* | | **0.962**  **0.000** | | | **1.000**  **0.000** | **1.000**  **0.000** | **0.948**  **0.000** | | **1.000**  **0.000** | **0.967**  **0.000** | **0.854**  **0.000** | **1.000**  **0.000** | | | **0.933**  **0.003** | | |
| P/BC | 0.000  1.000 | | 0.000  1.000 | 0.000  1.000 | | 0.000  1.000 | | *1.067* | | | 0.519  0.856 | 0.417  0.906 | 0.613  0.112 | | 0.686  0.126 | 0.487  0.506 | 0.581  0.153 | 0.562  0.663 | | | 0.647  0.775 | | |
| D/ID | 0.000  1.000 | | 0.000  1.000 | 0.000  1.000 | | 0.000  1.000 | | -0.007  1.000 | | | *1.300* | 0.361  0.784 | 0.750  0.402 | | 0.333  0.784 | 0.708  0.731 | 0.753  0.547 | 0.354  0.713 | | | 0.167  0.596 | | |
| CF/MT | 0.012  0.088 | | 0.018  0.478 | 0.018  0.457 | | 0.013  0.103 | | 0.001  0.524 | | | -0.033  1.000 | *0.760* | 0.732  0.069 | | 0.500  0.309 | 0.510  0.845 | 0.506  0.925 | 0.386  0.623 | | | 0.555  0.312 | | |
| BS/MT | 0.002  0.487 | | 0.002  0.596 | 0.002  0.614 | | 0.002  0.516 | | 0.002  0.486 | | | 0.002  0.720 | 0.012  0.076 | *1.280* | | 0.746  0.059 | 0.543  0.228 | 0.507  0.393 | 0.659  0.380 | | | 0.733  0.519 | | |
| E/AB | 0.000  1.000 | | 0.000  1.000 | 0.000  1.000 | | 0.000  1.000 | | 0.000  1.000 | | | 0.000  1.000 | 0.002  0.428 | 0.002  0.594 | | *1.130* | 0.583  0.628 | 0.692  0.262 | 0.273  0.865 | | | 0.648  0.126 | | |
| J/AB | 0.002  0.528 | | 0.002  0.610 | 0.002  0.603 | | 0.002  0.496 | | -0.002  1.000 | | | -0.008  1.000 | -0.003  0.548 | 0.004  0.183 | | 0.002  0.623 | *1.250* | 0.518  0.334 | 0.659  0.3996 | | | 0.690  0.707 | | |
| WC/AB | 0.000  1.000 | | 0.000  1.000 | 0.000  1.000 | | 0.000  1.000 | | -0.002  1.000 | | | -0.004  1.000 | 0.003  0.575 | 0.002  0.212 | | 0.000  1.000 | -0.001  0.603 | *1.265* | 0.746  0.169 | | | 0.869  0.033 | | |
| P/MB | 0.000  1.000 | | 0.000  1.000 | 0.000  1.000 | | 0.000  1.000 | | -0.006  1.000 | | | -0.037  1.000 | -0.174  1.000 | 0.002  0.629 | | 0.000  1.000 | -0.006  1.000 | -0.003  1.000 | *1.152* | | | 0.500  0.366 | | |
| PA/SK | 0.000  1.000 | | 0.000  1.000 | 0.000  1.000 | | 0.000  1.000 | | 0.000  1.000 | | | 0.000  1.000 | 0.026  0.478 | 0.002  0.728 | | 0.000  1.000 | 0.001  0.747 | 0.000  1.000 | 0.000  1.000 | | | *1.723* | | |

**Table S3.** Measures of differentiation between different groupings of populations of *D. recens* and *D. subquinaria*. Number of populations within each group is shown in parentheses. Within each species, the A groupings (sympatric versus allopatric populations) are the same as in Table 2 of the main text. Note that of the *F*_ST_ variants, *H*_ST_ is more useful for *D. recens COI* because it harbors low polymorphism, while *S*_nn_ is more useful for *D. subquinaria* because of its higher level of polymorphism [see Hudson et al. 1992 and Hudson 2000].

|  |  | AMOVA | | *F*_ST_ variants | |
| --- | --- | --- | --- | --- | --- |
|  | Source of Variation | % of variation | *F* | *H*_ST_ | *S*_nn_ |
| *D. recens* |  |  |  |  |  |
| A. | Regions: Allopatric (5) and sympatric (9) | -1.04 | Φ_CT_ = 0.010  (*P* = 0.662) | 0.007  (*P* = 0.409) | 0.539  (*P* = 0.048) |
|  | Among populations within regions | 13.88 | Φ_SC_ = 0.137  (*P* < 0.0001) |  |  |
|  | Within populations | 87.16 | Φ_ST_ = 0.128  (*P* < 0.0001) |  |  |
| B. | Regions: SM (1) and all others (13) | 32.78 | Φ_CT_ = 0.327  (*P* = 0.072) | 0.024  (*P* < 0.0001) | 0.897  (*P* < 0.0001) |
|  | Among populations within regions | 2.43 | Φ_SC_ = 0.036  (*P* < 0.0001) |  |  |
|  | Within populations | 64.79 | Φ_ST_ = 0.352  (*P* < 0.0001) |  |  |
| *D. subquinaria* |  |  |  |  |  |
| A. | Regions: allopatric (8) and sympatric (5) | 5.02 | Φ_CT_ = 0.050  (*P* = 0.172) | 0.000  (*P* = 0.617) | 0.601  (*P* = 0.027) |
|  | Among populations within regions | 22.82 | Φ_SC_ = 0.240  (*P* < 0.0001) |  |  |
|  | Within populations | 72.16 | Φ_ST_ = 0.278  (*P* < 0.0001) |  |  |
| B. | Regions: coastal (4) and inland (9) | 43.30 | Φ_CT_ = 0.437  (*P* < 0.0001) | -0.004  (*P* = 0.740) | 0.669  (*P* < 0.0001) |
|  | Among populations within regions | 0.38 | Φ_SC_ = 0.006  (*P* < 0.0001) |  |  |
|  | Within populations | 56.32 | Φ_ST_ = 0.433  (*P* < 0.0001) |  |  |
| C. | Regions: coastal (4), inland allopatric (4), and sympatric (5) | 31.00 | Φ_CT_ = 0.319  (*P* = 0.002) | 0.001  (*P* = 0.181) | 0.524  (*P* < 0.0001) |
|  | Among populations within regions | 0.66 | Φ_SC_ = 0.010  (*P* < 0.0001) |  |  |
|  | Within populations | 67.44 | Φ_ST_ = 0.325  (*P* < 0.0001) |  |  |
| D. | Regions: inland allopatric (4) and sympatric (5) | -0.55 | Φ_CT_ = -0.006  (*P* = 0.695) | -0.0003  (*P* = 0.441) | 0.525  (*P* = 0.691) |
|  | Among populations within regions | 0.17 | Φ_SC_ = 0.001  (*P* = 0.403) |  |  |
|  | Within populations | 100.38 | Φ_ST_ = -0.004  (*P* = 0.503) |  |  |
